# Beyond Traditional Silos: An Integrated Framework Leveraging Novel Pathways for Reducing Cardio-renal Risk in Type 2 Diabetes

**DOI:** 10.2174/0115733998345830250417094825

**Published:** 2025-05-29

**Authors:** Hani M. Sabbour, Mohamad Hassanein, Mohamed Farghaly, Harith M. M. S Aljubori, Nizar M. Attallah, Mona Al Rukhaimi, Mostafa Al-Ashkar

**Affiliations:** 1 Department of Cardiology, Mediclinic Hospital, Abu Dhabi, UAE;; 2 Department of Endocrinology and Diabetes, Dubai Hospital, Dubai, UAE;; 3 Postgraduate Diabetes Education, Cardiff University, Cardiff, UK;; 4 Dubai Medical College, Head of Insurance Medical Regulation, Senior Specialist at Dubai Health Authority, Dubai, UAE;; 5 Consultant for Nephrology and Transplantation, Al-Zahra Hospital, Sharjah, UAE;; 6 Consultant Nephrologist at Louisville, Nephrology Associates of Kentuckiana, Louisville, Kentucky, 40205, USA;; 7 Department of Medicine, Dubai Medical College, Dubai, UAE;; 8 Department of Nephrology, Dubai Hospital, Dubai, UAE;; 9 Private Researcher, UAE

**Keywords:** Chronic kidney disease, diabetes mellitus, cardiovascular disease, mineralocorticoid receptor antagonist, renin-angiotensin system, finerenone, sodium-glucose co-transporter-2 inhibitors

## Abstract

Diabetes mellitus is a major risk factor for chronic kidney disease (CKD) and accelerates kidney function decline, leading to end-stage renal disease. In addition, patients with CKD are at elevated risk of developing cardiovascular disease with its manifestations as coronary artery disease, heart failure, arrhythmias, and sudden cardiac death through the cardiorenal connection. An online regional and international expert panel of cardiologists, nephrologists, and diabetologists convened in November 2021 to obtain a broad perspective on the intersection of diabetes mellitus, CKD, and cardiovascular disease and identified treatment gaps that can help address the unmet needs of the patients in the Middle East region. The current review article summarizes the epidemiology, pathophysiology, diagnosis, and treatment options of CKD in diabetes and discusses the currently available treatment options to reduce morbidity and mortality in this high-risk population. The panel discussed the roles of clinical specialties and how to simplify the patient journey.

## 1 INTRODUCTION

Patients with chronic kidney disease (CKD) and type 2 diabetes mellitus have a significant impact on their cardiovascular (CV) health, which is well-established through the cardiorenal connection. This complex interaction of CKD pathophysiological characteristics, like activation of the renin-angiotensin system (RAS), fluid retention, or oxidative stress, leads to changes in the heart, such as ventricular hypertrophy or fibrosis. The recent approval of finerenone, a non-steroidal mineralocorticoid receptor antagonist (MRA) for the treatment of CKD associated with type 2 diabetes mellitus, has spurred interest as it is the first drug to target the disease-associated inflammation and fibrosis processes. New therapies such as SGLT2 inhibitors and finerenone have shown both renal and cardiovascular benefits in real- world patients.

## 2 OVERVIEW OF DIABETES BURDEN

The staggering pace at which diabetes is reaching epidemic proportions globally is alarming. According to the 2021 International Diabetes Federation’s (IDF) findings, around 537 million adults have diabetes. Moreover, diabetes was responsible for 6.7 million deaths, translating to almost one death per five seconds. Additionally, it was determined that 541 million adults exhibit impaired glucose tolerance, which increases their risk of developing type 2 diabetes mellitus. The number of people with diabetes is expected to reach 643 million by 2030 and 783 million by 2045 [1].

The prevalence of diabetes in the Middle East and North Africa region is equally disconcerting. According to the IDF’s 2021 statistics, 73 million (*i.e.*, 1 in 6 adults) in this region have diabetes, with this number projected to increase to 95 million by 2030 and 136 million by 2045. To add to these bleak findings, there is a possibility that 1 in 3 adults with diabetes may be undiagnosed [1].

Two major national surveys were conducted in the United Arab Emirates. The Weqaya Program, which included 50,138 participants, was conducted in Abu Dhabi. This program reported that 17.6% of the population was diagnosed with diabetes [2]. In another household survey, which was conducted in Dubai, revealed an overall prevalence of 13.7% of diabetes in Dubai. It also indicated that the prevalence of diabetes among Emiratis was 19.3% and among expats was 12.4% [3]. Further, 40.7% of the diabetic population in the region, which is still undiagnosed with diabetes, is heading us to an alarming situation [4].

## 3 DIABETES - A SILENT KILLER

Diabetes is known as the ‘silent killer’ for a valid reason. It slowly and stealthily brings a whole slew of complications that affect both smaller and larger vessels, *i.e.*, microvascular and macrovascular, respectively. The renal microvascular risk is the most expensive diabetic complication, with persistent kidney failure (nephropathy). Further, nerve injury (neuropathy) raises the likelihood of diabetic foot ulcers and/or amputations, and eye injury (retinopathy) can lead to blindness. Macrovascular disorders, such as coronary heart disease, peripheral artery disease, and stroke, significantly contribute to morbidity and mortality.

The Kidney Disease Improving Global Outcomes (KDIGO) guidelines define CKD as persistently elevated urine albumin excretion (≥30 mg/g [≥3 mg/mmol], persistently reduced estimated glomerular filtration rate (eGFR) of <60 ml/min per 1.73 m^2^), or both for more than 3 months. Diabetic kidney disease and diabetic nephropathy are both now called CKD [5, 6].

The rise of diabetes in the Middle East is likely to increase cardiac and renal complications. However, early diagnosis and multipronged intervention can improve prognosis and long-term patient outcomes.

## 4 PREVALENCE OF CKD IN DIABETES

According to the United States Renal Data System 2022 annual report, the prevalence of CKD in diabetes was 38.0%, compared to 10.4% in those without diabetes. Overall, 60.6% of incident patients with end-stage renal disease (ESRD) had diabetes mellitus [7].

As per the Imperial College London Diabetes Centre 2019 report, nephropathy was reported as a diabetes-related complication in 87.95% of adults (≥18 years only) [8].

The results from Dialysis Outcomes and Practice Patterns Study showed that diabetes mellitus in Bahrain, Kuwait, Oman, Qatar, Saudi Arabia, United Arab Emirates ranged from 45% to 74% in patients with hemodialysis by country. Overall, 76% of patients with diabetes also had diabetes listed as their primary cause of ESRD [9].

A meta-analysis was conducted on the studies published between 2009 and 2018 to evaluate CKD prevalence in diabetes mellitus in the Middle East. The studies collectively included 59,395 participants with a mean age range of 50.8 to 66.9 years. The analysis revealed the CKD prevalence in diabetes mellitus (types 1 and 2) to be between 10.8% and 60.78%, with the pooled prevalence being 28.96%. Selection, screening, reporting biases, type of clinical practice- primary, secondary, or tertiary healthcare setting, hospital location, and medical specialty explain the enormous range observed in this analysis [10]. More epidemiological studies and stringent screening protocols can provide a better estimate of the true prevalence of CKD in diabetes mellitus in the Gulf region.

All these statistics underscore the indisputable link between diabetes and CKD. Moreover, diabetes is also a major risk factor for cardiovascular disease (CVD), thereby prompting the American Diabetes Association (ADA) to recommend intensive CV risk factor management in diabetes patients [11, 12]. CKD increases the risk of CVD in the form of coronary artery disease, particularly heart failure, arrhythmias, and sudden cardiac death [13]. The European Society of Cardiology (ESC) designates that the presence of diabetes mellitus confers a two-fold excess risk of vascular outcomes (coronary heart disease, ischemic stroke, and vascular deaths), independent of other risk factors. Further, both absolute and relative risk levels of vascular events become higher in those with long-standing diabetes mellitus and microvascular complications, including renal disease or proteinuria [14]. Thus, the presence of both CKD and diabetes further amplifies the CVD risk and predicts poor outcomes, including increased mortality [11, 14].

## 5 CKD IN DIABETES: RISK FACTORS

While heart disease, obesity, a family history of CKD, inherited kidney disorders, past kidney damage, and older age are all predisposing factors for CKD, diabetes and hypertension are the most important risk factors. Moreover, diabetes and hypertension are often comorbid conditions.

Risk factors include [15]:

- Susceptibility factors:
  - Age, sex, race/ethnicity, and family history.
- Initiation factors:
  - Hyperglycemia, acute kidney injury, nephrotoxic drugs, infections.
- Progression factors:
  - Hypertension, dietary factors, and obesity.

## 6 PATHOPHYSIOLOGY OF CKD IN DIABETES

Pathophysiology of CKD in diabetes involves the convergence of multiple hemodynamic (elevated blood pressure [BP] and/or intraglomerular pressure), metabolic (inadequate glycemic control), inflammatory and fibrotic processes. These processes collectively lead to tubulointerstitial damage, inflammation, mesangial expansion, glomerular hypertrophy, and glomerulosclerosis contributing to kidney fibrosis and CKD progression [15].

The hemodynamic pathway involved in the pathogenesis is driven by RAS activation, which increases angiotensin II levels. Increased angiotensin II results in efferent arteriolar vasoconstriction, increased albuminuria, and nephropathy [16]. Significant metabolic changes in early diabetes alter kidney hemodynamics and trigger inflammation and fibrosis. These changes include hyperglycemia and hyperaminoacidemia, which promote glomerular hyperfiltration and hyperperfusion [15].

The mineralocorticoid receptor (MR) is a nuclear receptor and is present in various tissues, including the kidney and heart. Activation of MR induces transcription of genes involved in electrolyte homeostasis and tissue remodeling. However, overactivation of MR upregulates the expression of genes involved in inflammatory and fibrotic processes like NADPH oxidase, pro-inflammatory cytokines, and pro-fibrotic mediators (Fig. **1**) [17]. The complex interaction with pathophysiological characteristics of CKD, like activation of the RAS, fluid retention, or oxidative stress, leads to changes in the heart, such as ventricular hypertrophy or fibrosis [13].

Guided by the pathophysiology, the mainstay therapy of CKD in diabetes for many years has involved managing hyperglycemia and controlling BP using renin-angiotensin-aldosterone system (RAAS) inhibitors [18]. Multiple studies, like Reduction of Endpoints in NIDDM with the Angiotensin II Antagonist Losartan (RENAAL) and Irbesartan Diabetic Nephropathy Trial (IDNT), have demonstrated the positive impact of RAAS inhibition on cardiorenal outcomes. RAAS inhibition protects the kidneys by lowering intraglomerular pressure and reducing hyperfiltration [18].

The MR pathway represents a novel therapeutic target in CKD pathogenesis. Ligands that bind the MR can block the MR overactivation and curb the downstream inflammatory and fibrotic processes, thereby conferring renoprotection and cardio-protection by slowing down CKD progression [17].

## 7 EFFECTS OF CKD ON CARDIOVASCULAR HEALTH: CARDIORENAL CONTINUUM

Owing to the strong cardiorenal connection, CKD patients are at an increased risk of CVD, which commonly presents as heart failure with preserved ejection fraction (HFpEF) with left ventricular hypertrophy (LVH) and diastolic dysfunction. The LVH prevalent in 50%-70% of CKD patients can further lead to heart failure with reduced ejection fraction (HFrEF), arrhythmias, ischemic heart disease, and sudden cardiac death [19].

The CV mortality accounts for 40% to 50% of all deaths in patients with advanced CKD stages 4 and 5 [20]. There is extensive overlap in the regulation and functioning of the renal and cardiac systems. The RAAS, the sympathetic nervous system, natriuretic peptides, endothelin, and antidiuretic hormones have a role in the homeostatic functioning of the two systems. A dysfunction in the cardiac system affects the renal system and vice versa. Even a mild to moderate kidney function deterioration can translate to higher morbidity and mortality in high-risk CVD patients. Thus, CKD and CVD are interlinked conditions that share common risk factors like dyslipidemia, smoking, obesity, inflammation & oxidative stress, iron deficiency, hypoxia, pharmacotherapy, and pathophysiological processes encompassing the cardiorenal continuum [21].

Further, compared to CKD or type 2 diabetes mellitus alone, concomitant type 2 diabetes mellitus and CKD amplify the risk of CV mortality almost two to three times (Fig. **2**) [22]. The link between CKD and CV mortality implies that the therapies limiting renal damage can also positively impact CV outcomes.

Reduced quality of life and increased risk of premature CV mortality in CKD significantly impact the socio-economic and healthcare burden associated with diabetes [23].

A large meta-analysis was conducted on data collected from more than one million participants, which included 130,000 participants with diabetes. The analysis results indicated that the risk of CV mortality increased with an increasing level of albuminuria and decreasing eGFR (Fig. **2**). Chronic kidney disease patients showed a higher risk of cardiovascular mortality, irrespective of diabetes status. Although the absolute risk of CV mortality is higher in CKD patients with diabetes than those without, the relative risk is similar in both cohorts [24]. Thus, assessment of both albuminuria and eGFR, which are independent risk factors for CKD and CV mortality, is required to ensure timely diagnosis and proactive management to prevent adverse outcomes. These parameters are inadequately screened at the primary care level and by cardiologists, resulting in delayed diagnoses and subsequent specialty referrals, adversely affecting patients’ long-term outcomes.

A subgroup study on Heart Outcomes Prevention Evaluation trial participants to examine the relationship between baseline albuminuria levels and CVD risk in patients with or without diabetes mellitus demonstrated that any degree of albuminuria is a risk factor for CV events with or without diabetes [25].

## 8 DIAGNOSIS OF CKD

Diagnosis of CKD in its initial stages is challenging as it is not associated with any overt symptoms. Although a definitive diagnosis of diabetic glomerulopathy requires a kidney biopsy, routine annual screening of diabetic patients can help diagnose CKD even without a biopsy [12].

Annual uACR and eGFR tests are recommended for all type 2 diabetes patients, with additional testing for those at higher risk of progression. These assessments are also crucial before making any vital therapeutic decisions [6, 14, 26].

Increased urinary albumin excretion can be screened by assessing uACR in a random spot urine collection. Timed or 24-hour collections are not recommended as they are not significantly more accurate than spot urine samples [27].

## 9 RESIDUAL RISK OF CKD PROGRESSION

The RAS blockers like angiotensin-converting enzyme inhibitors (ACEis) or angiotensin II receptor blockers (ARBs), primarily developed as antihypertensive drugs, slow down CKD progression. However, data from studies like RENAAL [28] (on losartan) and IDNT [29]. (on irbesartan) show that despite cardioprotective and reno-protective effects of RAS blockade, the residual risk of CKD progression exists in patients with comorbid CKD and type 2 diabetes mellitus.

In the RENAAL and IDNT studies, nearly 43.5% and 21% of patients, respectively, experienced the primary renal endpoint (doubling of the serum creatinine concentration, ESRD, or death) despite treatment with losartan and irbesartan, highlighting the large magnitude of residual risk [28, 30].

A large systematic review and meta-analysis of 13 SGLT2is trials was conducted on data collected from 90,413 participants (82.7% participants with diabetes and 17.3% without diabetes included in the heart failure and CKD studies). The results of meta-analysis revealed that overall, compared with placebo, allocation to an SGLT2i reduced the risk of kidney disease progression by 37%. However, in the 4 chronic kidney disease trials (CREDENCE, SCORED, DAPA−CKD, EMPA−KIDNEY), the overall relative risk for the kidney failure was 0.67 (0.59-0.77) (Fig. **3**) [31].

In the CKD patient population, death due to CV events is more likely than progression to ESRD, which represents the natural pathophysiological endpoint of CKD [32]. Notably, reduction in kidney function (reduced eGFR) and presence of kidney damage (albuminuria) are independent risk factors for CV mortality in the general and high-risk populations, irrespective of other traditional CV risk factors [33, 34]. Compared to optimal eGFR levels, the mortality risk becomes statistically significant in the general population at an eGFR of around 60 ml/min/1.73 m^2^, and an exponential increase in mortality risk is observed at even lower eGFR values. This observation is independent of the albuminuria status. Additionally, the relationship between albuminuria and mortality is linear on the log-log scale (2-fold increase at uACR of ~100 mg/g compared to an optimal level of 5 mg/g) and independent of the eGFR level [34].

Thus, the high residual risk of disease progression translating to increased CVD mortality highlights the urgent need for additional therapies to further reduce this risk and improve long-term renal and CV outcomes.

Reducing the risk of CKD progression thus calls for a comprehensive strategy to control glycemia, BP, and lipids. Despite optimal therapies, high residual risk remains, highlighting the need to utilize novel treatments that have recently been shown to address this residual risk by targeting alternate pathophysiological processes.

## 10 MANAGEMENT OF CKD IN TYPE 2 DIABETES MELLITUS PATIENTS: SUMMARY OF TREATMENT OPTIONS AND GUIDELINE RECOMMENDATIONS

Treatment decisions are personalized depending on the risks of complications and potential clinical outcomes.

Mainstay therapies for CKD include those that alter the hemodynamic and metabolic factors driving CKD progression. Long-term glycemic and BP control are vital to reducing the rate of CKD progression (Fig. **4**).

The KDIGO guidelines recommend treating diabetes patients with hypertension or albuminuria with ACEis or ARBs. They recommend maintaining a BP target below 120/80 mmHg using standardized BP measurements in all patients [35]. The KDIGO guidelines also recommend considering an ACEi or ARB in patients with diabetes and albuminuria but normal BP, owing to the strong correlation between the severity of albuminuria and the risk of kidney failure [6], based on the results from RENAAL [27] and INNOVATION [36] trials, which included normotensive participants and showed a reduction in uACR levels with RAAS inhibition [6].

For glycemic control in diabetes patients, stringent glycosylated hemoglobin (HbA1c) targets may be considered in young patients with a shorter duration of diabetes, absence of complications, and longer life expectancy. Less stringent HBA1c targets may be considered in older patients with a longer duration of diabetes, microvascular and macrovascular complications, and limited life expectancy [6]. The guidelines recommend using metformin and an SGLT2i as first- line treatment for glycemic control. Long-acting glucagon- like peptide-1 receptor agonists (GLP1-RAs) are recommended in type 2 diabetes mellitus patients with CKD who cannot reach glycemic targets with metformin and SGLT2is or who cannot use these interventions due to intolerances or any contraindications [6].

The ESC 2023 guidelines recommend intensive low-density lipoprotein lowering with statins or a statin/ezetimibe combination to reduce CV risk. To reduce kidney risk, ACEi or ARB with the maximum tolerated dose is recommended. For type 2 diabetes patients with CKD and eGFR ≥20 ml/min/1.73 m^2^, recommend an SGLT-2i and aim for a BP target of 130/80 mmHg to reduce CV and kidney risk [14].

The updated KDGIO guidelines suggest using a nonsteroidal MRA in patients with type 2 diabetes mellitus, an eGFR ≥25 ml/min per 1.73 m^2^, normal serum potassium concentration, and albuminuria (≥30 mg/g [≥3 mg/mmol]) despite maximum tolerated dose of RAS inhibitor, is based on the results of Finerenone in Reducing Kidney Failure and Disease Progression in Diabetic Kidney Disease (FIDELIO-DKD) and Finerenone in Reducing Cardiovascular Mortality and Morbidity in Diabetic Kidney Disease (FIGARO-DKD) trials which included patients with type 2 diabetes mellitus with albuminuria and had serum potassium levels less than 4.8 mmol/l at screening. Both trials examined the CV and kidney effects of finerenone [6].

The most recent 2023 ESC and ADA guidelines also support the KDIGO guidelines and recommend the use of nonsteroidal MRA in addition to other medications for CV and kidney protection rather than as alternatives when other treatments fail [14, 37].

The consensus of endocrinologists, cardiologists, and nephrologists experts in this panel, is that multipronged interventions are needed to address the metabolic and cardiorenal effects of diabetes, particularly in the presence of CKD. They emphasize lifestyle alterations, weight loss, and salt restriction to improve the prognosis of patients with diabetes and CKD. In high-risk CVD patients, lowering low-density lipoproteins with the addition of a statin may be necessary. Statins can also help lower albuminuria (PLANET I and II) [38]. In addition, screening for exceedingly early stages of renal disease can improve prognosis and long-term patient outcomes. The importance of comprehensive cardio-renal or cardio-metabolic clinics was discussed. The consensus was that the utilization of disease-modifying therapies such as SGLT2is in diabetes is suboptimal despite availability in the region. All the experts agreed on the need for evidence-based therapies with novel mechanisms of action that can address residual risk and further reduce the rate of CKD progression and the risk of CV events.

## 11 RECENTLY APPROVED THERAPIES ON THE HORIZON

Despite RAS blockade and SGLT-2 inhibition, there is still a substantial risk of CKD progression. The currently available therapeutic agents in the CKD armamentarium target metabolic and hemodynamic processes but do not address inflammation and fibrosis, which is a very promising target pathway. Although the intricacies of the inflammatory and fibrotic processes involved in CKD are still being unraveled, the role of MR overactivation as a key trigger in these processes has been well-established. Thus, drugs blocking MR overactivation have now been shown in outcome randomized clinical trials (RCTs) to address the residual risk of CKD progression and improve long-term patient CV outcomes.

Drugs blocking MR activation belong to two categories—steroidal and non-steroidal MRAs.

Non-selective steroidal MRAs in clinical practice are currently used in heart failure and resistant hypertension only, and not in widespread use in CKD due to concerns about hyperkalemia, especially in CKD patients [30, 39].

Steroidal MRAs, spironolactone and eplerenone, demonstrate antihypertensive, cardioprotective, and antiproteinuric effects even at low doses. These agents block the effects of aldosterone on tubuloglomerular feedback, endothelial damage, and fibrosis. The risk of hyperkalemia has prevented long-term trials on spironolactone and eplerenone in high-risk CKD patients, limiting their clinical use [40].

Safer non-steroidal MRAs that mimic the effects of steroidal MRAs are in development. The non-steroidal MRAs, finerenone, apararenone, AZD9977, KBP-5074, and esaxerenone are in different stages of clinical development [41].

Compared to steroidal MRAs, non-steroidal MRAs have the absence of active metabolites, a shorter half-life, and balanced distribution between the heart and kidney. All these factors favor less risk of hyperkalemia.

## 12 FINERENONE - TARGETING FIBROSIS AND INFLAMMATION ADDRESSES A MAJOR COMPONENT OF RESIDUAL RISK WITH A NOVEL EVIDENCE-BASED MECHANISM

Two large phase III RCTs examined the CV and kidney effects of finerenone: FIDELIO-DKD [42] and FIGARO-DKD [43]. Two trials in 48 countries included over 13,000 type 2 diabetes mellitus patients with various levels of CKD severity. A prespecified pooled analysis of the FIDELIO-DKD and FIGARO-DKD trials, named FIDELITY [44], was published.

The FIDELIO-DKD [42] data showed that finerenone reduces the risk of CKD progression and CV events in patients with type 2 diabetes mellitus and advanced CKD. Similarly, in FIGARO-DKD [43], compared to placebo, finerenone improved CV outcomes in type 2 diabetes mellitus patients with stage 2-4 CKD and moderately elevated albuminuria or stage 1-2 CKD with severely elevated albuminuria. These results demonstrate the beneficial renal and CV effects of finerenone in patients with advanced CKD and type 2 diabetes mellitus. Moreover, the side-effect profile of finerenone was like that of the placebo, albeit with a low absolute risk of clinically relevant hyperkalemia.

FIDELITY has further validated the positive CV and renal outcomes of finerenone in a broad spectrum of CKD patients, suggesting a possible additive effect between finerenone and SGLT-2is or GLP-1RAs that may be attributed to their distinct mechanisms of action [45]. However, as these observations are from a prespecified pooled analysis, additional RCTs will be needed to assess the safety and efficacy of SGLT2-inhibitor/finerenone and GLP-1RA/ finerenone combinations in CKD treatment [45] (Fig. **5**).

Advanced CKD, type 2 diabetes mellitus, and ACEis or ARBs therapy increase hyperkalemia risk. In the FIDELIO-DKD trial, hyperkalemia was reported in the treatment/finerenone arm was 11.8% *vs* 4.8% in the placebo arm. The maximum mean difference in serum potassium between these 2 groups was 0.23 mmol/l in the 4^th^ month. Further, clinically relevant hyperkalemia leading to hospitalization was reported only for 1.4% in the treatment arm *vs* 0.3% for the placebo arm. Physicians can mitigate this increased risk by monitoring serum potassium and managing hyperkalemia with the strategy used in the FIDELIO-DKD trial [46]. However, it should be noted that the cardiorenal benefits observed in the overall patient population also extended to those with the highest hyperkalemia risk.

Because of the combined cardiorenal benefits of finerenone and underlying RAS blockade, continuation of finerenone with utilization of potassium binders (RAS enablement) would be recommended to reduce CV events.

Despite recommendations in several guidelines, MRAs remain underused in CVD patients with CKD due to a higher risk of adverse events in this population [47]. According to the experts on this panel, less than 1% of patients with hypertension and ~50%-60% of those with heart failure are treated with MRAs due to hyperkalemia risk and renal dysfunction concerns. The availability of selective non-steroidal MRAs like finerenone will allow physicians to overcome the therapeutic inertia against their use and make their application in primary care and across different specialties more feasible.

The KDIGO 2022 guidelines include a class 2A recommendation for the use of non-steroidal MRAs in type 2 diabetes mellitus patients with an eGFR ≥25 ml/min/1.73 m^2^, normal serum potassium concentration, and albuminuria despite a maximum tolerated dose of a RAS inhibitor [6].

Recent updates in ADA guidelines recommend that patients with type 2 diabetes mellitus and CKD should be considered for treatment with finerenone to reduce CV outcomes and the risk of CKD progression [37].

This is furthered strengthened by data from FIDELITY [45] analysis where in 6.7% of patients who were on SGLT-2is showed reduction in uACR by 37% with addition of finerenone, thus, reflecting additive effects of the therapy.

In this context, it is essential to note that finerenone, a novel non-steroidal MRA, with proven clinical cardiorenal benefits in type 2 diabetes mellitus patients with CKD is a new paradigm in the treatment of these patients.

## 13 OTHER PATHWAYS CURRENTLY IN CLINICAL TRIALS FOR CKD

New drugs will continue to be discovered as our understanding of the pathogenic mechanisms of CKD progression improves.

Pirfenidone is an anti-fibrotic agent that interrupts the TGF-β pathway. The role it plays in delaying CKD progression is currently being evaluated [48]. Pentoxifylline, which modulates inflammatory and oxidative stress-related responses, has been shown to reduce proteinuria when added to RAS blockade in CKD patients [49].

## 14 CHALLENGES IN THE MANAGEMENT OF CKD IN TYPE 2 DIABETES MELLITUS

Undoubtedly, there is an urgent need to develop new therapeutic interventional strategies. Nonetheless, the CKD patient journey can also be simplified if the commonly encountered challenges are addressed.

CKD is asymptomatic in the initial stages, making it difficult to diagnose. The metabolic derangement is observed only during the later stages [10]. CKD screening protocols can be further optimized in the Gulf region to facilitate early diagnosis and intervention, thereby preventing disease progression.

Persistent increased microalbuminuria (uACR of 30-299 mg/g) has been identified as an early marker for CKD development and increased risk of CVD in type 2 diabetes mellitus. However, microalbuminuria screening is requested very rarely in cardiology departments, representing a significant gap in clinical practice that needs to be addressed. The experts on the panel agreed that more frequent screening of uACR (*i.e.*, every 6 months, instead of annually) could help diagnose CKD in its initial stages and improve prognosis. It was recognized that all practitioners that GFR and albuminuria are highly effective biomarkers at predicting CVD risk and they both should be universally utilized.

Elevated microalbuminuria with normal to mildly reduced eGFR is an indicator of CKD and CVD risk, and so is normal albuminuria with moderately to severely reduced eGFR [50]. In the classical model of CKD in type 2 diabetes mellitus disease progression, the onset of moderate-to-severely increased albuminuria precedes an eGFR decline (below 60 ml/min/1.73 m^2^). However, albuminuria does not necessarily progress linearly and can vary over the entire course of disease progression. Regression to normoalbuminuria may also occur in some patients [15, 51]. Due to such a heterogeneous nature of the disease, some patients present with reduced eGFR without or before developing albuminuria [52]. As many cardiologists do not evaluate GFR, except in specific scenarios such as when they need to intensify therapy in heart failure, and before cardiac catheterization to determine the risk of contrast nephropathy, the opportunities for early CKD detection and intervention are lost.

Primary care physicians and cardiologists do not screen patients for uACR as often as they should, thereby delaying CKD diagnosis and referral to appropriate specialists.

Diagnostic and therapeutic inertia are prevalent in certain spheres of clinical care and often lead to suboptimal treatment. At times, physicians fail to set appropriate goals or are reluctant to modify treatment to achieve the set treatment goals. There is scope for better control of hypertension with the available therapies (either by dose optimization or adding-on treatment) in patients with diabetes, CKD, and proteinuria. It is also important to highlight that physicians find it challenging to give enough time to patients due to extremely crowded outpatient units and a shortage of support staff [53]. While managing CKD patients, cardiologists should use GFR and microalbuminuria as biomarkers of CV risk.

Chronic kidney disease (CKD) impacts cardiovascular, metabolic, and renal health. An interdisciplinary approach can enhance the management of this condition. However, there are only a limited number of multidisciplinary clinics in the region that include nephrologists, endocrinologists, and cardiologists. Easy access to multidisciplinary clinics is a significant unmet need in the CKD treatment landscape. All healthcare centers should have cardiorenal metabolic clinics for comprehensive diabetes care for CKD patients. These clinics may have specialized nurses instead of diabetes educators. Access to such clinics would ensure that the patients are timely and comprehensively evaluated.

Recent clinical trials have shown that SGLT2is may benefit patients with a lower eGFR threshold than initially recommended. Due to frequent updates in evidence, the approved label has undergone multiple modifications, reducing the GFR thresholds from 60 to 20 ml/min/1.73 m^2^. This modification is frequently disregarded by medical professionals. Previous guidelines advise against initiating these drugs if eGFR is below 60 ml/min/1.73 m^2^, excluding patients with proteinuria who might benefit. Therefore, physician updates are necessary. Regulatory authorities should update package inserts based on recent clinical trial evidence.

Polypharmacy in CKD patients reduces patient compliance with prescribed therapies.

According to the experts on this panel, most patients are aware of CKD. Still, they need additional education and counselling to better understand their disease prognosis and the risks of progression. Certain patients are unable to modify their lifestyle and dietary habits, potentially worsening their prognosis.

Despite widely available insurance coverage, there is a difference in tiers of insurance. In addition, different insurance companies have different policies regarding annual screening covering GFR and ACR.

## 15 DISCUSSION

We should take a comprehensive approach to managing diabetes and its complications.

The updated KDIGO and ADA guideline consensus states ‘A nonsteroidal MRA with proven kidney and CV benefit is recommended for patients with type 2 diabetes mellitus, an eGFR ≥25 ml/min/1.73 m^2^, normal serum potassium concentration, and uACR ≥30 mg/g despite maximum tolerated dose of RASi.’

Further, 2023 ESC guidelines recommends use of finerenone in addition to an ACEi or ARB in patients with type 2 diabetes mellitus and eGFR >60 mL/min/1.73 m^2^ with a uACR ≥30 mg/mmol (≥300 mg/g), or eGFR 25-60 mL/min/1.73 m^2^ and uACR ≥3 mg/mmol (≥30 mg/g) to reduce CV events and kidney failure.

Therefore, the first step towards effective management is standardizing annual screening for GFR and ACR. Robust screening of diabetes complications like CKD would lead to early intervention and improved outcomes. Fool-proof screening of CKD at the primary care level requires educating the primary care physicians about the current clinical practice guideline recommendations on screening protocols. The primary care physicians should also be made aware of the link between CKD and CVD death risk to ensure that they screen patients carefully and refer them to appropriate specialists as early as possible. Establishing multidisciplinary groups (like cardiometabolic, renal-metabolic, and cardiorenal groups) is the need of the hour that will help provide patients with proper specialist care and allow specialists to collaborate effectively while simultaneously garnering real-world clinical experience with novel therapies like finerenone. Lack of published data from the region highlights the importance of establishing national and regional cardio-renal metabolic registries that could initially be started within the well-established large diabetes center in each country.

## CONCLUSION

Finerenone is an effective treatment that addresses a major component of CKD residual risk by targeting fibrosis and inflammation. Data from phase 4 studies on finerenone would provide additional insights into its impact on the real-world CKD patient population. 6.7% of patients enrolled in the finerenone trials were on SGLT2is, which showed additive effects of both therapies. Although mechanistic studies and the data from FIDELITY analysis show that SGLT2is and finerenone may have an additive effect, whether it also applies to the real-world CKD patient population remains to be seen. The RCTs on finerenone/SGLT2i combination therapy may shed more light on the extent of this potential additive effect. Nevertheless, based on the data from FIDELIO-DKD and FIGARO-DKD, it can be concluded that there was no negative interference between finerenone and SGLT2is.

Developing a CKD therapy targeting inflammation and fibrosis has taken decades, tapping into a previously unused mechanism in CKD treatment. The selectivity of finerenone for the MR will potentially allow its broader clinical application in multiple disease areas beyond CKD in type 2 diabetes mellitus. Studies to look at the role of finerenone in controlling CKD from etiologies other than type 2 diabetes mellitus are currently underway.

## Figures and Tables

**Fig. (1):**
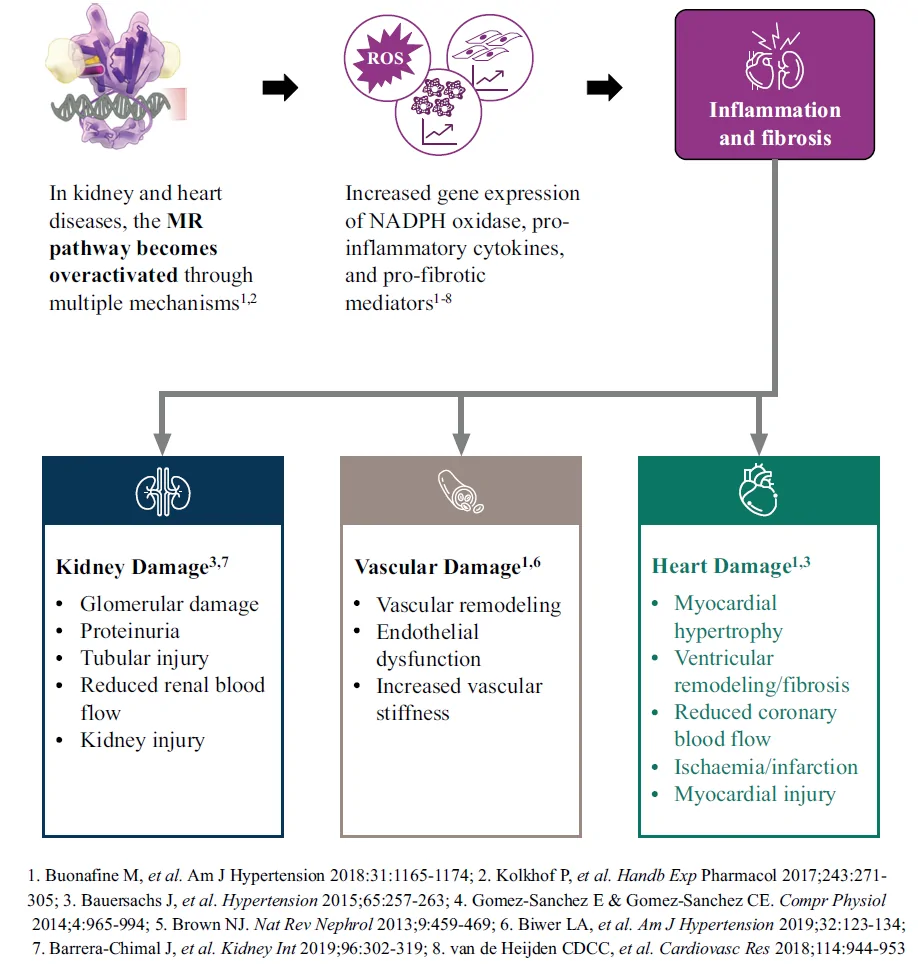
MR overactivation leading to kidney and cardiovascular damage.

**Fig. (2):**
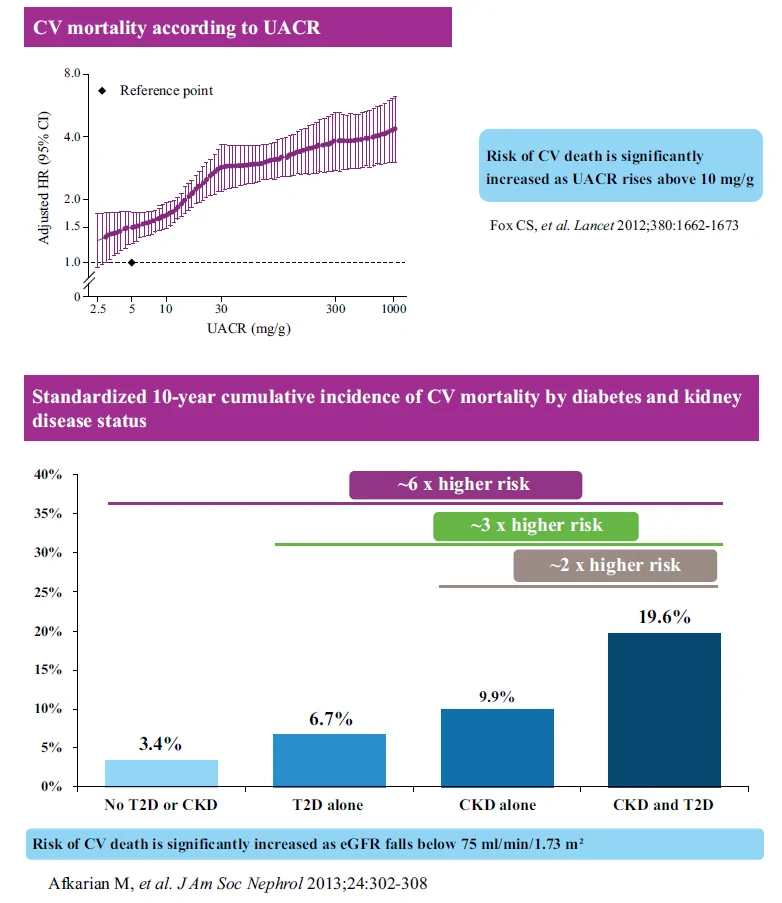
Increased risk of CV events in diabetic patients.

**Fig. (3):**
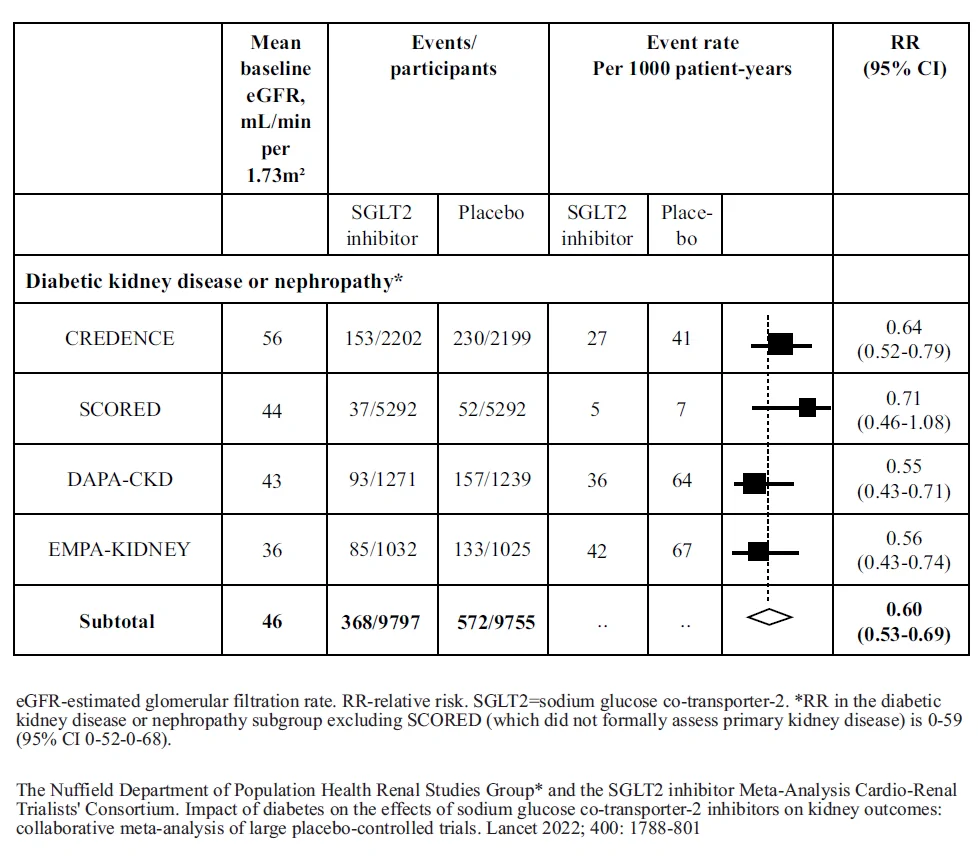
Effect of sodium glucose co-transporter-2 inhibition on kidney disease progression.

**Fig. (4):**
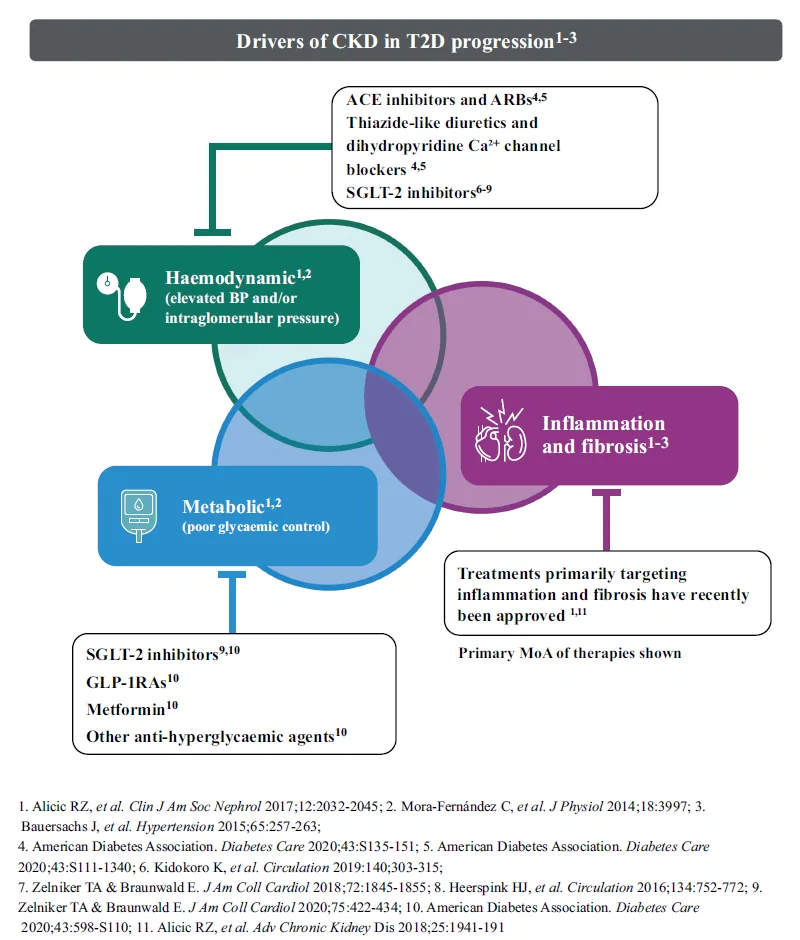
Current CKD therapies.

**Fig. (5):**
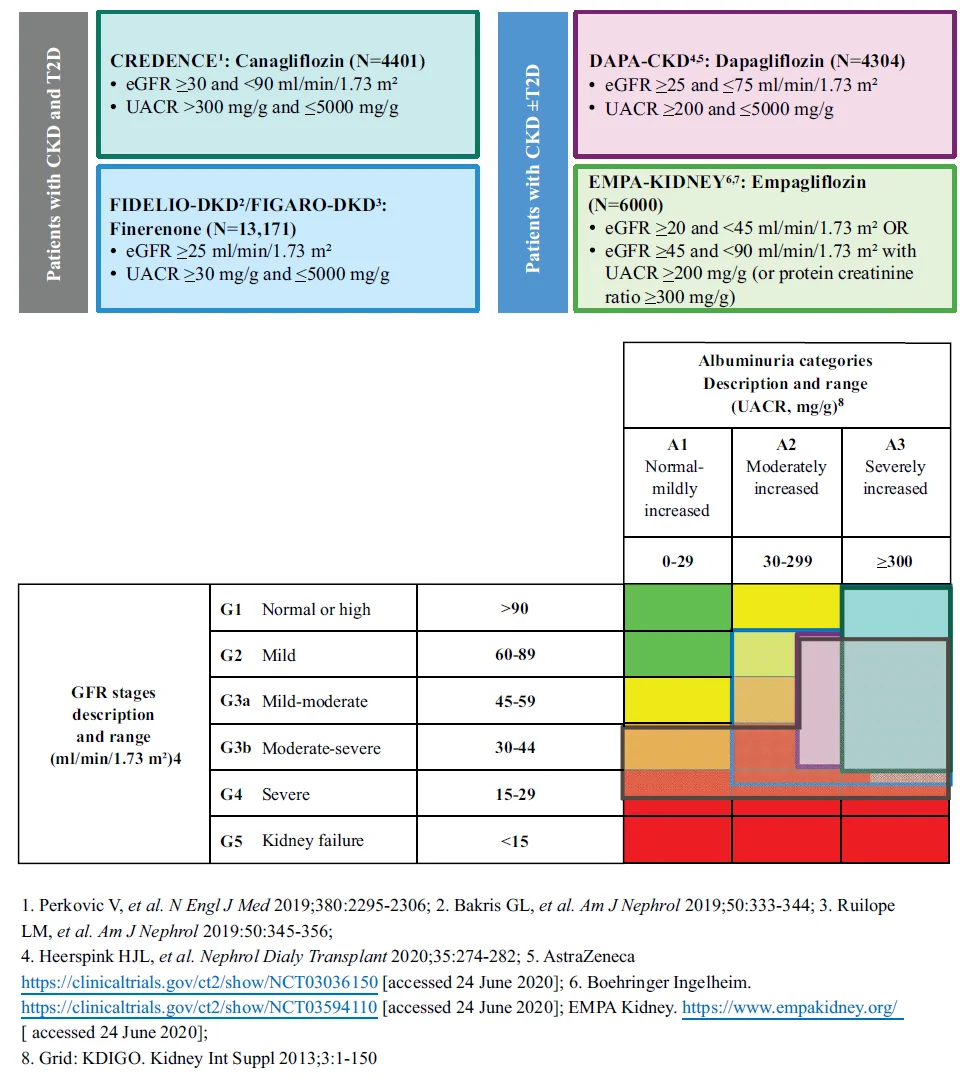
Finerenone results in CKD patients with type 2 diabetes mellitus.

## LIST OF ABBREVIATIONS

BP: Blood Pressure
ESC: European Society of Cardiology
CVD: Cardiovascular Disease
ADA: American Diabetes Association
ESRD: End-Stage Renal Disease
KDIGO: Kidney Disease Improving Global Outcomes
